# Microneedles Based on a Biodegradable Polymer—Hyaluronic Acid

**DOI:** 10.3390/polym16101396

**Published:** 2024-05-14

**Authors:** Jagoda Chudzińska, Agata Wawrzyńczak, Agnieszka Feliczak-Guzik

**Affiliations:** Faculty of Chemistry, Adam Mickiewicz University in Poznań, Uniwersytetu Poznańskiego 8, 61-614 Poznań, Poland; jagoda.chudzinska@amu.edu.pl (J.C.); agaguzik@amu.edu.pl (A.F.-G.)

**Keywords:** microneedle, hyaluronic acid, transdermal delivery system

## Abstract

Transdermal transport can be challenging due to the difficulty in diffusing active substances through the outermost layer of the epidermis, as the primary function of the skin is to protect against the entry of exogenous compounds into the body. In addition, penetration of the epidermis for substances hydrophilic in nature and particles larger than 500 Da is highly limited due to the physiological properties and non-polar nature of its outermost layer, namely the *stratum corneum*. A solution to this problem can be the use of microneedles, which “bypass” the problematic epidermal layer by dispensing the active substance directly into the deeper layers of the skin. Microneedles can be obtained with various materials and come in different types. Of special interest are carriers based on biodegradable and biocompatible polymers, such as polysaccharides. Therefore, this paper reviews the latest literature on methods to obtain hyaluronic acid-based microneedles. It focuses on the current advancements in this field and consequently provides an opportunity to guide future research in this area.

## 1. Introduction

The transdermal transport of active substances is an alternative to traditional drug delivery routes, such as intramuscular or intravenous injections and oral administration [1]. It is viewed as an effective and easy-to-use way of distributing drugs and active substances. The challenge is to discover new methods that allow hydrophilic and hydrophobic substances to penetrate the skin effectively. The answer may lie in bypassing the outer layer of the epidermis, namely the *stratum corneum* (SC), and such a solution is offered by microneedles, which are able to pierce the epidermis, but their size is so small that the application is virtually painless [2]. In addition, such a carrier of active substances can be made of compounds that are biocompatible and have a positive effect on the skin, which is an additional advantage [3].

## 2. Microneedles

The use of transdermal drug delivery systems (TDDSs) such as microneedles allows the non-invasive and virtually painless dosing of active ingredients [4]. Microneedles (MNs) are small protrusions with sizes between 25 μm and 2000 μm. They are used to penetrate the epidermis and release the active ingredient in the appropriate layer, depending on the length of the protrusions. In addition, they can be an alternative to the traditional injections, creams, and ointments routes for delivering active substances into deeper layers of the skin [5]. Due to the wide application of this type of active substances’ transportation, several types, shapes, and materials of which microneedles can be composed have been established.

### 2.1. Types of Microneedles

**Figure 1:**
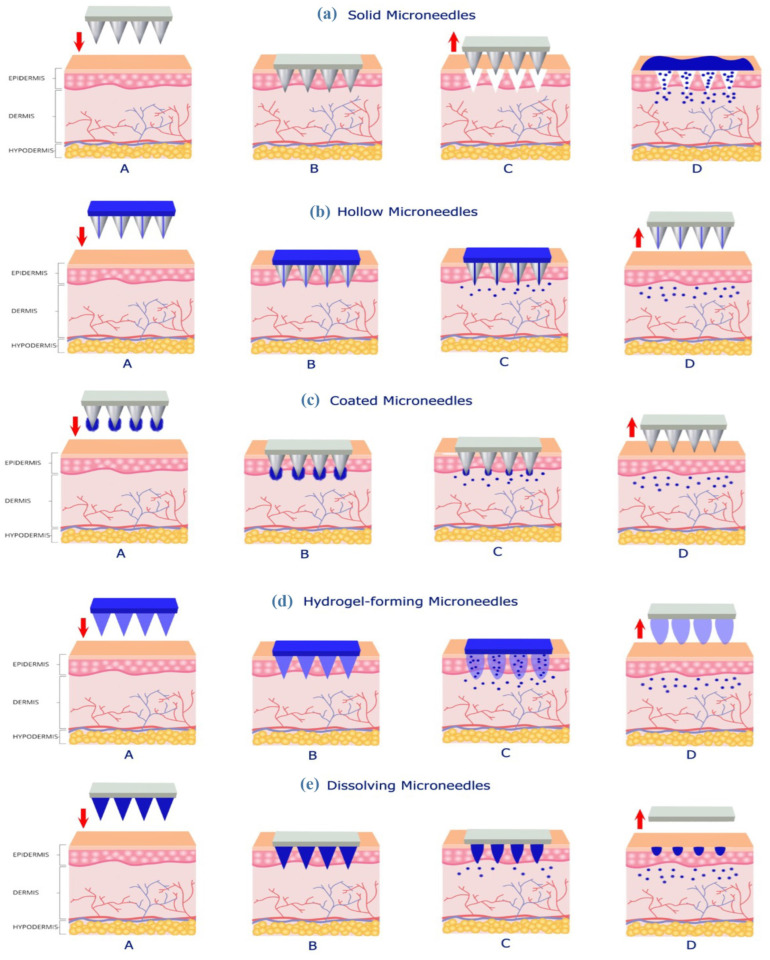
Schematic representation of the release mechanism of active substances from different types of microneedles: (**a**) solid; (**b**) hollow; (**c**) coated; (**d**) hydrogel; (**e**) soluble; A—insertion of microneedles into the *stratum corneum*; B-C—release of active substances; D—removal of microneedles from the *stratum corneum*; red arrows indicate direction of the movement of the microneedles patch [6] (Published by Elsevier B.V. This is an open access article under the CC BY license).

Several types of microneedles can be distinguished. They differ in their construction and mechanism of the active substance release and include (i) solid, (ii) hollow, (iii) coated, (iv) hydrogel, and (v) soluble microneedles (Figure 1).

#### 2.1.1. Solid MNs

Solid microneedles are entirely filled with the material from which they have been synthesized. Their task is to create microchannels in the skin. Then, after the removal of microneedles, a cream, ointment, or gel containing an active substance is applied to the surface of the skin, and it can penetrate into the previously created microchannels (pores) (Figure 1a) [7,8].

#### 2.1.2. Hollow MNs

Hollow microneedles have a solid skeleton with a hollow chamber/core in the center, which is filled with an active substance in the form, for example, of a suspension or solution. At the end of each spear, there is an opening through which the active substance is delivered directly to the skin (Figure 1b). The pressure and dispensing rate of the active substance can be adjusted by changing, among others, the size of the hole at the end of the spear. This type of microneedle is characterized by the ability to dispense large amounts of a drug and coils of large sizes [5,7,8].

#### 2.1.3. Coated MNs

Microneedles of this type consist of a solid matrix, where the protrusions are coated with a layer of active substance in the form of a solution or dispersion. The active substances are released from the surface of the needles after application to the skin (Figure 1c). The amount of drug delivered can be controlled by changing the thickness of the coating layer and the length of the needles [9,10,11]. It is possible to deliver several different active substances with a single microneedle patch; in this case, each needle is coated with a different active substance [12].

#### 2.1.4. Hydrogel-Forming MNs

This type of microneedles is prepared from insoluble polymers that swell and crosslink in the presence of water, forming a hydrogel. After a hydrogel microneedle patch is applied, the protrusions absorb water in the form of interstitial fluids and swell. The mechanism of the active ingredient release is based on the diffusion through the swollen MNs matrix into the dermal tissue (Figure 1d). This type of MNs exhibits the ability to load more drugs than other types of microneedles [7,13,14].

#### 2.1.5. Dissolving MNs

Dissolvable microneedles are made of water-soluble, biodegradable materials. The active ingredient is dispersed in the matrix and released gradually as the needle dissolves in the skin (Figure 1e) [5,7,15]. This type of MNs generates the least waste due to the fact that it is completely or mostly (if only the tabs are soluble) dissolved. The release of the active ingredient from the soluble microneedles can occur rapidly or slowly, depending on the polymer used [16,17]. The limitations of soluble microneedles are related to the mechanical strength of the tips, which must penetrate the *stratum corneum* [18].

### 2.2. Shapes of Microneedles

Microneedles may have various shapes and forms. The design of a microneedle patch includes [19]:

- matrix—a flat element that carries the needles and remains on the surface of the skin;
- microneedles—protruding elements (protrusions), which, depending on the application, can have a length up to 2000 μm, diameter up to 250 μm, and a tip thickness of 1–25 μm [19].

The microneedles can be hollow or completely filled, as in the case of solid, soluble, or hydrogel microneedles [20]. A single needle can have different shapes, specifically cylindrical, conical, pyramidal, and pentagonal, with a pointed or cylindrical tip [19]. The tips can also take other forms, such as funnel or candle-like (with a taper at a certain height of the needle). Moreover, the needle tip may be a circle, triangle, square, or star-shaped. The shape of the needle affects the penetration capacity of the skin and the amount of active substances delivered [21,22,23].

### 2.3. Materials for Microneedles Preparation

#### 2.3.1. Metal

Metal alloys or pure metals, such as stainless steel, titanium, nickel, tantalum, and palladium, are most commonly used for the preparation of microneedles. These materials exhibit good mechanical properties and general biocompatibility. The disadvantage is their lack of elasticity and biodegradability. They work well in obtaining solid, coated, and hollow microneedles [11,24,25].

#### 2.3.2. Ceramic

Ceramic is a porous solid material that consists of inorganic compounds of metals and non-metals. The porosity can be freely modified during the manufacture of this raw material. It has good strength, chemical resistance, and biocompatibility, while being a good thermal and electrical insulator [11,25,26].

#### 2.3.3. Silicon and Glass

Silicon is a crystalline, hard, and brittle solid. Silicon compounds are used, among others, in the medical industry in the form of silicon glass or silicon. Materials based on silicon compounds have good chemical and thermal stability [11,25,27].

#### 2.3.4. Polymer

Polymers are created by a large group of compounds that can be used in the production of microneedles. Synthetic and natural polymers (so-called biopolymers) can be distinguished. Polymers used to obtain microneedles should be characterized by high biocompatibility and biodegradability, as well as high strength and the ability to allow the appropriate penetration and continuous release of active ingredients. Depending on the polymer used, it is possible to design microneedles of almost any type. However, polymers are used mainly in the production of soluble and hydrogel microneedles [4,28,29,30]. 

Among the macromolecular compounds that have been successfully used for microneedles synthesis are water-soluble sugars (e.g., sucrose, mannitol, xylitol, trehalose, and galactose) and biodegradable polymers, including poly(lactic-glycolic acid) (PLGA), polylactic acid (PLA), polycaprolactone (PCL), polyvinyl alcohol (PVA), polyvinylpyrrolidone (PVP), carboxymethylcellulose (CMC), poly(methyl methacrylate) (PMMA), silk fibroin, and 3-aminophenylboronic acid-modified alginate or hyaluronic acid (HA). The penetration of microneedles through the skin is the biggest obstacle for polymeric microcarriers, as unlike insoluble materials (e.g., silicon or copper), the mechanical strength of water-soluble polymers is often lower, and encapsulation of the active ingredient can further weaken their strength [31,32].

In general, polymers are used to produce three categories of long-acting microneedles, specifically biodegradable, swellable, and both. Natural, semi-synthetic, and synthetic polymers can be used alone or in combination with other compounds. They can be found in all three categories of MNs and allow the appropriate period of drug release [30,32]. Biodegradable polymers are almost insoluble in the interstitial fluid of the skin, but they slowly degrade in the skin tissue. Thus, the release of the active substance is mainly controlled by degradation of the polymer matrix rather than by diffusion of the drug. Consequently, drug release profiles from MNs can be easily controlled by modifying the physicochemical properties of polymers, namely their molecular weight and the molar ratio of the particular blocks [33]. In contrast, swelling polymers are neither soluble nor degradable in skin tissue. They swell by absorbing interstitial fluid in the skin and form hydrogels. The drug release pattern is based mostly on the passive diffusion of the active ingredient molecules, which is influenced by the solubility of the active ingredient, the crosslinking density of the polymer, and the interaction between the polymer and the active ingredient. Microneedles obtained from swelling polymers should be removed after application, which affects the safety issues with respect to polymer residues and degradation products. Polymers from natural sources (such as silk fibroin and gelatin) are both biodegradable and swellable. The release of the active ingredient from these types of polymers is more complex than those presented earlier, as it is affected by both the swelling ability and the rate of the polymer degradation [33].

Microneedles based on one of the biopolymers, namely hyaluronic acid, will be described in more detail further in the article.

## 3. Hyaluronic Acid

Microneedles based on polymers with high biodegradability, biocompatibility, and solubility are the subject of interest of many researchers, and their popularity is still growing [31,32]. Polymer-based microneedles allow the design of carriers with rapid or prolonged (72 h to 14 days) release of the active substance [33]. As mentioned above, polymers are a large group of compounds in which we can distinguish synthetic polymers from biopolymers. Biodegradable compounds of synthetic origin include PLA—poly(lactic acid), PLGA—poly(lactic-co-glycolic) acid, and PCL—polycaprolactone [33,34,35]. Biopolymers are compounds of natural origin, and this group mainly includes polynucleotides, polypeptides, and polysaccharides [36,37].

Polysaccharides are common natural polymers that are derived from renewable sources such as plants, algae, fungi, or bacteria. Popular polysaccharides include chitosan, alginate, and hyaluronic acid [38]. Due to widespread occurrence, polysaccharides are commonly used as biomaterials for a variety of applications, including the production of microneedles [39].

**Figure 2:**
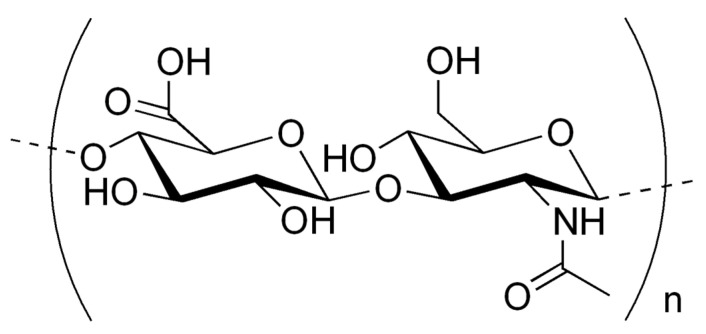
Chemical formula of hyaluronic acid.

Hyaluronic acid (HA) belongs to the group of glycosaminoglycans (known as GAGs) made up of disaccharide units (Figure 2). HA is a long unbranched polysaccharide composed of repeating mers of D-glucuronic and N-acetyl-D-glucosamine [40,41].

This biopolymer can be obtained from various sources: it is synthesized by animal hyaluronan synthases (HASs), and then, it can be isolated by extraction from animal tissues [42]. It is also possible to obtain HA by microbiological methods, including bacterial fermentation [43].

Hyaluronic acid can be divided by molecular weight using its medium ranges [44,45,46]:

- low molecular weight hyaluronic acid (LMW-HA) 0.5–1.5 × 10^6^ Da;
- medium molecular hyaluronic acid (MMW-HA) 1.2–6 × 10^6^ Da;
- high molecular weight hyaluronic acid (HMW-HA) 3–7 × 10^6^ Da.

Depending on the source, HA can have different molecular weights. Examples of sources of hyaluronic acid and its respective molecular weights are shown in Table 1 [47].

Hyaluronic acid is continuously synthesized and degraded in the human body. The degradation process can be called depolymerization, as the glycosidic bonds between the N-acetyl-D-glucosamine and D-glucuronic acid residues are broken. In humans, HA degradation can proceed by two mechanisms: specific enzymatic degradation and non-specific radical degradation. Both mechanisms summarily degrade about 30% by weight of the total HA content in the human body. The remaining 70% is systematically catabolized with HA transported to the lymph nodes through the lymphatic system, where it is degraded by endothelial cells of lymphatic vessels [48].

The most important characteristics that make hyaluronic acid often used for microneedling is its high biocompatibility, biodegradability, durability, lack of toxicity, and strong hygroscopic properties. Moreover, relative to other common polysaccharides (e.g., chitin, chitosan, and alginate), hyaluronic acid is a major extracellular component of connective tissue that plays a significant role in lubrication, cell differentiation, and proliferation. In addition, the structure of the main chain of this compound contains functional groups, such as carboxylic and hydroxyl, which can be used to introduce other functional groups or to crosslink hydrogels. Hyaluronic acid’s unique mechanical properties allow it to maintain tissue hydration, which benefits cell infiltration [49].

In addition, hyaluronic acid of different molecular weights can have different effects on the skin. HMW-HA has anti-aging properties, forms a film on the skin’s surface, and retains moisture. MMW-HA and LMW-HW can penetrate deeper into the skin, performing moisturizing actions [46]. As a result of a number of desirable properties, hyaluronic acid is an attractive base material for the transdermal transport of active ingredients using microneedles.

Hyaluronic acid is commonly used in cosmetics as a moisturizing agent. Furthermore, HA-based microneedles can be enriched with additional active substances, such as peptides, which will have a beneficial effect on the appearance and condition of the skin. It is also used to treat skin diseases such as psoriasis, melasma, and wounds. HA-based microneedles are also beginning to be used for drug delivery in cancer therapies [50]. On the other hand, alginate and hyaluronic acid-based microneedles, produced by the micro-molding technique, can be used for transdermal insulin delivery in type 1 diabetes. They showed sufficient mechanical strength for skin penetration, as well as good degradability [32].

## 4. Fabrication of Hyaluronic Acid-Based Microneedles

Hyaluronic acid-based microneedles are an object of interest due to the properties of HA, but the methods for preparing these carriers are quite limited. Currently, methods such as micro-molding, photolithography, and centrifugal lithography are commonly used [51]. However, methods based on additive technology, 3D printing, and droplet-born air blowing are also gaining popularity.

### 4.1. Micro-Molding

This method is one of the most popular ways of preparing hyaluronic acid-based microneedles. The preparation procedure involves filling the mold with a suspension or diluent previously prepared with the active substance [52]. Several micro-molding techniques can be identified, including micro-injection and solvent-casting.

Micro-injection molding (µIM) is one of the mold-filling techniques with the advantages of replication accuracy and a short filling cycle time. This process involves injecting a liquid material into the mold cavity at high speed and an elevated temperature (if the material used is solid at room temperature). The parameters of the microneedles obtained depend on the type of mold used for casting [53,54].

**Figure 3:**
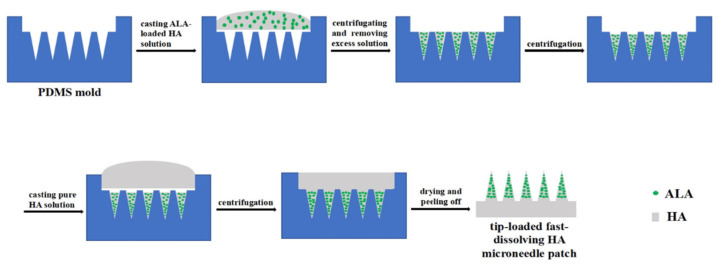
Schematic illustration of the MNs fabrication process using the micro-molding technique (HA—hyaluronic acid and ALA—active substance). Reprinted from [58], Copyright (2018), with permission from Elsevier.

The most commonly used technique is micro-molding by solvent-casting. It is due to a simple procedure that can be performed at room temperature [55]. The solvent-casting method is used mainly to produce soluble microneedles based on biodegradable, water-soluble polymers, such as hyaluronic acid [56]. The preparation of a microneedle patch begins with the preparation of a solution containing HA and an active ingredient. This process depends on the compound with which the microneedles are loaded. Then, in order to remove bubbles and completely fill the tips of the microneedles, the mold (usually made of silicone with PDMS—polydimethylsiloxane) with the solution/suspension is subjected to centrifugation, vacuum, reduced pressure, or the freezing and thawing procedure. The mold with the solution is then dried by evaporating the solvent to maintain the microneedles, which, after drying, can be released from the mold (Figure 3) [57].

Table 2 presents the most recently developed hyaluronic acid-based MNs prepared using the micro-molding method.

### 4.2. Droplet-Born Air Blowing

**Figure 4:**
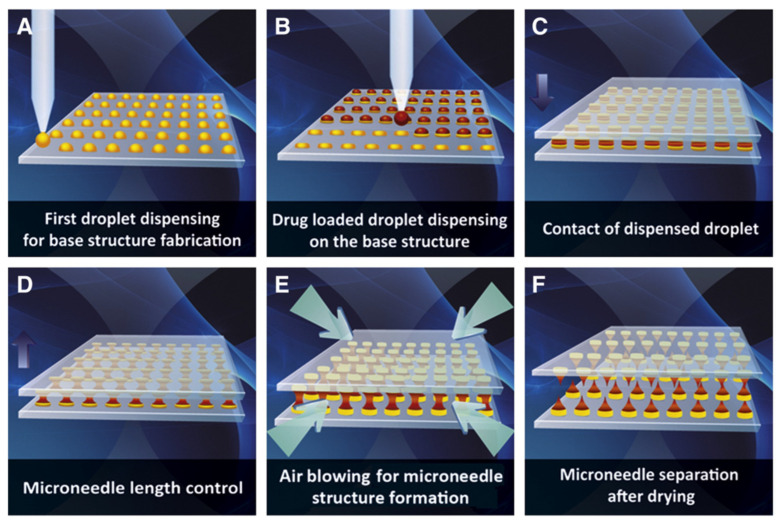
Schematic illustration of dissolving MNs fabrication via the droplet-born air blowing method. (**A**) Biopolymer dispensing on the flat surface for base structure fabrication. (**B**) Dispensing of drug-containing droplet over the base structure. (**C**) Contact of dispensed droplet by downward movement of upper plate. (**D**) Control of microneedle length. (**E**) Air blowing-mediated solidification of droplet to shape microneedle structure. (**F**) Separation of two plates producing dissolving microneedle arrays on upper and lower plate. Reprinted from [80], Copyright (2013), with permission from Elsevier.

Another method used to prepare microneedles is droplet-born air blowing (DAB). It involves the formation of polymer droplets using air blowing (Figure 4). The process takes place at an ambient temperature in the range of 4–25 °C and is very short (about 10 min). An important aspect is the viscosity of the polymer diluent; it should be viscous enough to form a needle structure between two plates [77]. This method allows the formation of microneedles in a one-step procedure without the need for high temperatures or UV radiation. However, the disadvantage of this method may be the lack of reproducibility [78].

The sequence of this method is based on [79]:

- applying single drops of a polymer solution to a flat wafer surface;
- dispersing droplets loaded with an active substance across the wafer surface;
- overlapping of two plates—the point of contact is the previously applied drop;
- controlled upward movement of the upper plate, which allows adjustment of the length of the microneedle;
- air blowing, which solidifies the droplets formed earlier;
- plate separation, which allows the recovery of the microneedles formed.

In 2019, the research group of Avcil produced hyaluronic acid-based microneedles containing arginine/lysine polypeptide, acetyl octapeptide-3, palmitoyl tripeptide-5, and adenosine, as well as seaweed (*Undaria pinnatifida* and *Corallina officinalis*), extracts. They used the DAB method, in which each droplet was blown with air to solidify and form the microneedle. A 12-week clinical study confirmed the positive effects of the prepared microneedle patches, which improved the thickness and density of the dermis and increased skin hydration [81].

### 4.3. Photolithography

**Figure 5:**
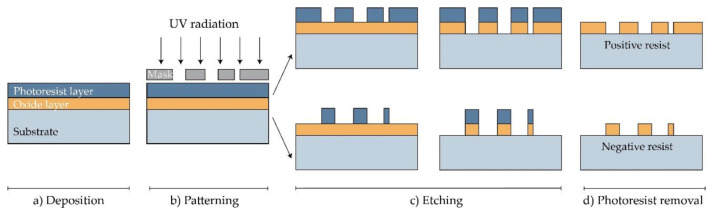
Production of MN by photolithography. (**a**) Deposition: the substrate is treated with steam or undergoes hydration to form an oxide layer, then the photoresist material is coated onto the silicon substrate (wafer) by spin coating. (**b**) Patterning: UV light shines through the mask onto the polymer. (**c**) Etching: the soluble polymer is removed, and the silicon layer is etched. (**d**) Photoresist removal. Reprinted from [85] (MDPI Open Access).

Photolithography is a technique that is used mainly to produce microneedles from ceramics and polymers. It allows the production of both solid and hollow MNs. This method involves coating a silicon substrate (wafer/plate) with a photosensitive polymer by spin coating. Then, the polymer, called photoresist, is thermally cured and exposed to UV light through a special mask, which consists of UV-impermeable material, so that the desired pattern is generated on the silicon wafer. Ultraviolet rays act on the polymer’s bonds by initiating or inhibiting crosslinking. The part of the polymer that has been exposed to UV radiation differs considerably in solubility from the one covered by the mask. The part of the photoresist not covered by the mask is then eliminated by immersing the substrate in the appropriate solution. The final step is the etching process, which involves reproducing the solid made of photoresist on the substrate by a dry or wet chemical attack [82,83,84]. Figure 5 shows schematically the microneedle fabrication process using photolithography.

The research group of Chen and Zhang [86,87] in 2022 proposed a process to produce hyaluronic acid-based microneedles by photolithography. This was accomplished by using modified HA, namely methylacrylated hyaluronic acid (HAMA) containing a 5% multi-thiol bioactive crosslinking agent (SH-CA). The esterification reaction was carried out with pentaerythritol and N-acetyl-l-cysteine to form this crosslinking agent (SH-CA). Due to the combination and reaction between HAMA and SH-CA, it was possible to cure the hyaluronic acid-based gel with UV light [86,87].

A rapid method for producing microneedles using heating and photolithography was presented by the research team of Kathuria. A microneedle patch containing hyaluronic acid as the model active ingredient was prepared by pre-polymerization of the N-vinylpyrrolidone solution at elevated temperatures, followed by the application of a photolithography procedure. After the initial polymerization of N-vinylpyrrolidone (heating for 2 min at 90 °C), the polymer was cooled for 1 min at room temperature. Then a 20% *w*/*v* or 40% *w*/*v* hyaluronic acid solution was added to the PVP matrix. The prepared mixture was poured into an oiled PDMS mold, which was further subjected to vacuum and cured with UV light. The study showed that pre-polymerization and heating allowed a reduction in the UV exposure time, and thus, the total preparation time was also reduced [88].

### 4.4. Centrifugal Lithography

**Figure 6:**
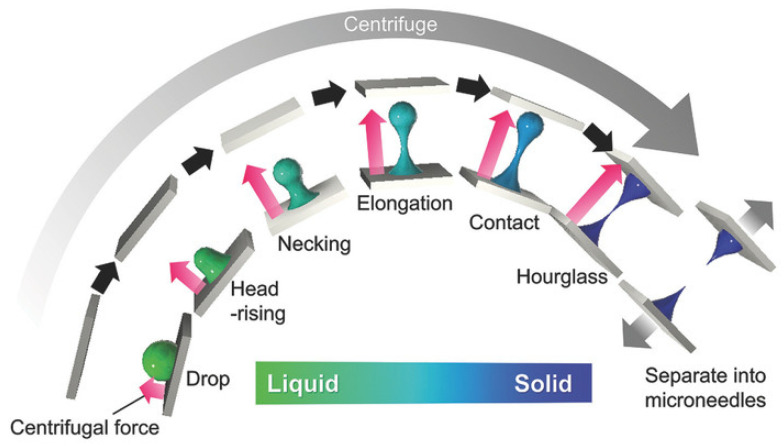
Schematic representation of the formation of soluble microneedles based on hyaluronic acid using the centrifugal lithography method. Reprinted from [90]. Copyright (2017), with permission from John Wiley and Sons.

Centrifugal lithography (CL) uses centrifugal force to form microneedles from a viscous polymer stratum [89]. It is a one-step method that does not require additional environmental factors such as heat, UV radiation, or air blowing, and thus, it is suitable for preparing carriers with temperature- or radiation-sensitive active compounds. Figure 6 shows the process of microneedle formation using the CL method [90].

In 2023, the research group of Juhng obtained a three-layer microneedle containing hyaluronic acid-based liraglutide (TLM) by centrifugal lithography. The preparation of the microneedles consisted of the application of a base layer (hyaluronic acid solution), followed by a layer containing the encapsulated active ingredient, and a final protective layer of hyaluronic acid solution. The use of three layers allowed the active substance to be protected from environmental stress and completely incorporated into the skin. The prepared TLM patch allowed painless application and long-lasting release of the drug. A study found that daily application of this formulation reduced body weight and body composition in a mouse model of obesity induced by a high-fat diet [89].

In turn, Jang’s research group applied centrifugal lithography to prepare soluble microneedle patches based on high and low molecular weight hyaluronic acid (HMW-HA and LMW-HA) and containing adenosine (Ad). The prepared solutions of adenosine and hyaluronic acid at a ratio of 1:600 were homogenized, dispensed with an array of 25 droplets onto a carboxymethylcellulose (CMC) film using a dispensing robot, and subjected to the centrifugal lithography procedure. Finally, the CMC film containing the dissolving microneedles matrix was placed on a viscous hydrocolloid patch, resulting in the formation of Ad-HMN and Ad-LMN. The obtained Ad-HMN and Ad-LMN formulations were subjected to 12-week clinical efficacy and safety tests, which confirmed the improvement in skin elasticity and density, as well as the reduction of wrinkles. Although both the Ad-HMN and Ad-LMN formulations showed positive effects, the high molecular weight HA-based microneedles showed better results [91].

### 4.5. Three-Dimensional Printing/Additive Manufacturing

The 3D printing method, also known as the additive method (AM), is currently of interest to many research groups. The additive production of microneedles (3D printing) allows complete control over their composition [92]. The use of Computer-Aided Design (CAD) software makes it possible to design microneedles with specific lengths and geometries that can be produced with high reproducibility [17]. Various techniques of 3D printing are used to produce microneedles, namely [93,94]:

- stereolithography (SLA);
- selective laser sintering (SLS);
- digital light processing (DLP);
- fused deposition modeling (FDM);
- two-photon polymerization (2PP);
- continuous liquid interface production (CLIP).

The application of the above-mentioned technologies makes it possible to obtain different types of polymer-based microneedles. Table 3 summarizes the 3D printing technologies and their application in the production of microneedles [93,95].

3D printing can be used to produce both microneedles and molds. However, microneedle formation still has many limitations due to the limited variety of materials that can be used in this technology [101].

Among the additive manufacturing methods, the SLA technique was used to produce microneedles based on biodegradable polymers, resulting in MNs with high quality and strength. This study showed that it is possible to prepare microneedles from biodegradable polymers using the SLA technique and opened up new possibilities for the design and production of modern MNs [102].

On the other hand, a study by Galarraga’s research group presented the possibility of preparing a hydrolysable hyaluronic acid-based hydrogel that can be used as a resin in the DLP technique [103]. In this study, norbornene-modified HA (NorHA_CA_), which is susceptible to thiol-ene crosslinking, was used for the synthesis. Post-reaction extraction with carbic anhydride was used to form hydrolytically unstable networks. NorHA_CA_ has been synthesized with variable degrees of modification, ranging from 15% to 100%. The degree of modification has been adjusted by the ratio of the reactive carbic anhydride to the amount of HA. This material was then reacted under visible light in the presence of a crosslinking agent (dithiol) and a photoinitiator, resulting in a hydrogel obtained within seconds. Thus, synthesized materials have been implemented as resins in DLP bioprinting to produce, among others, hydrolytically degradable scaffolds [103].

## 5. Future Directions

The preparation and application of microneedles is still a topic that offers many possibilities. Similarly, hyaluronic acid-based carriers can be involved in many functions. As evidenced by the recent increase in the number of publications on this topic, scientific groups around the world are constantly working on new technologies for obtaining microneedles with high quality, good mechanical properties, and the expected release profile of the active substance [104].

One of the most interesting directions in the use of microneedles is the treatment of oral ulcers. Researchers of the Zeng group designed and manufactured a microneedle patch containing an anti-inflammatory substance, namely dexamethasone (DXMS) and angiogenic basic fibroblast growth factor (bFGF), which were added to a hyaluronic acid solution and placed at the tips of the microneedles. In vivo tests showed that the microneedles effectively inhibited inflammation and promoted angiogenesis. In addition, the matrix based on hyaluronic acid and hydroxypropyltrimethylammonium chitosan chloride (HACC) showed antibacterial activity [59].

The scientific group of Terashima presented a procedure for obtaining hollow microneedles, where the core was made of PLA (polylactic acid) while the inner material was a 5% *w*/*w* gel created by mixing sodium hyaluronate with distilled water [105]. In general, the latest advances in microneedle synthesis have led to microneedles formed from hydrogels that not only have the ability to transdermally deliver drugs but also, due to the swelling nature of the hydrogel, to passively draw interstitial fluid from the skin, becoming potential monitoring devices characterized by high biocompatibility and minimal invasiveness [13].

Recent studies have also described the preparation of matrix-separating hydrogel microneedles based on hyaluronic acid with niacinamide for the treatment of skin pigment deposition. The microneedles were prepared by a solvent-free solid-state crosslinking method based on HA, with niacinamide as the active ingredient. A solution of hyaluronic acid and poly(vinyl methyl ether)-maleic acid (GAN), containing the active substance, was poured over the very ends of the needles. The matrix that separated after application was prepared with polyvinylpyrrolidone (PVP). Thus, the prepared microneedle patch combined the advantages of soluble and hydrogel microneedles [106].

The research team of Huang proposed the synthesis of soluble microneedles based on hyaluronic acid that release the drug upon activation at a certain pH. These microneedles can be applied in the combination chemophotodynamic therapy of melanoma. The DHA@HPFe-MN patch was created by crosslinking at the tip of the needle a protoporphyrin (PpIX)-ADH-hyaluronic acid (HA) conjugate (HA-ADH-PpIX) with a PA-Fe^3+^ complex, along with dihydroartemisinin (DHA), which was incorporated into the hydrogel network. The acidic tumor microenvironment activates Fe and DHA; therefore, the prepared patch allows the controlled release of the active ingredient [107].

Another interesting study was performed by Hao and co-workers, who obtained soluble microneedles that respond to near-infrared radiation [68]. By loading 5-fluorouracil (5-Fu) and indocyanine green (ICG) on the monomethoxy-poly(ethylene glycol)-polycaprolactone (MPEG-PCL) nanoparticles, the 5-Fu-ICG-MPEG-PCL system was obtained. Subsequently, 5-Fu-ICG-MPEG-PCL was integrated with hyaluronic acid-based soluble microgels (HA MNs) to obtain a material for the treatment of skin cancers, including, but not limited to, melanoma. Studies in mice have shown promising results, confirming the anti-tumor properties of the above-mentioned microneedles [68].

Another important aspect of the future use of microneedles is diagnostics, especially health monitoring. MNs integrated with appropriate sensors quite often use fluorescent or electrochemical reactions. These sensors can be placed on the backside of the MN array or along the MN tips. MN-based monitoring devices, specifically those obtained with biopolymers such as hyaluronic acid, have the potential to revolutionize health diagnostics by providing rapid and almost non-invasive tests outside traditional laboratories [108]. Zheng et al. reported the synthesis of a biosensor based on methacrylated hyaluronic acid (MeHA) MNs with a fluorescently labeled aptamer probe. This sensor reveals a rapid and simple approach to link aptamer probes to the MeHA matrix of MNs and provides a reagentless fluorescence assay by capturing and detecting specific biomarkers. The ex vivo tests demonstrated the possibility of using MeHA MNs patches functionalized with aptamer probes in the highly sensitive and specific detection of glucose, adenosine triphosphate, l-tyrosinamide (a mimic of l-tyrosine), and thrombin. Moreover, they were proven useful in tracking fluctuations in the glucose levels in an animal model of diabetes, with some parameters dominant over those in commercially available glucose monitoring devices [109]. Takeuchi et al. developed a technique to obtain the porous PDMS MNs array coated with hyaluronic acid by using the salt leaching and mold-casting method. The in vivo test showed that the obtained biosensor can be inserted into mouse skin and easily extract interstitial fluid to measure the glucose concentration. It offers a new concept of flexible HA MNs for continuous health monitoring [110].

Undoubtedly, one of the most noteworthy future directions in MN production is the use of artificial intelligence (AI), both in the classical synthesis methods and those based on 3D printing. This will pave the way for the next generation of MNs. The review paper by Biswas and co-workers pointed out many aspects in this area, particularly the possibility of using AI to predict drug release patterns, as well as being a quality control tool to assess MNs obtained by 3D printing [111].

The integration of AI algorithms with continuous monitoring technologies, such as continuous glucose monitoring (CGM), has the potential to revolutionize chronic disease management and personalized healthcare. CGM biosensors based on microneedles and closed-loop control AI algorithms are gaining popularity due to reduced pain and tissue damage compared to the traditional monitoring tools. In addition, electrochemical devices based on microneedles made out of biocompatible materials, such as HA, could simultaneously monitor glucose levels and implement diabetes therapy by delivering the appropriate drugs. This would bridge the gap between data collection and analysis and improve the therapeutic accuracy of microneedle-based devices [112]. Luzuriaga et al. conducted a study on the possibility of obtaining biodegradable microneedles by fused deposition modeling (FDM) 3D printing. For this purpose, they used a biodegradable and FDA-approved polymer, namely, polylactic acid. By applying AI-based 3D modeling software, MNs with personalized shapes, densities, and lengths were obtained, with the ability of various degrees of penetration into the outer layers of the skin and delivery of a model therapeutic agent [113].

## 6. Conclusions

Microneedle patches are an innovative way of delivering active substances by the transdermal route. Non-invasiveness and easy application make them suitable for self-dosing by patients. Biodegradable, soluble microneedles, which do not generate medical waste that is difficult to dispose of, seem to be an interesting solution. The use of hyaluronic acid for this purpose brings up additional advantages, such as biocompatibility and availability of the raw material. In addition, the matrix of microneedles based on hyaluronic acid provides opportunities for the incorporation of many active substances with therapeutic potential against various skin conditions (e.g., psoriasis and melanoma) or systemic diseases (e.g., Alzheimer’s disease). Therefore, multifunctional polysaccharide-based composite MN patches can be considered as an innovative and promising solution among transdermal drug delivery systems.

## Figures and Tables

**Table 1:** Examples of HA from different sources and their molecular weights [47].

Source of HA	Molecular Weight [kDa]
Rooster combs	1200
Umbilical cord	3400
Bovine vitreous body	770–1700
Bacteria	1000–4000
Human joint fluid	6000–7000
Rheumatoid fluid	3000–5000

**Table 2:** Hyaluronic acid-based microneedles prepared by micro-molding, their application, and their preparation procedure.

Sample Labeling	MNs Material	Active Substance	Mold Material	Synthesis Procedure	Potential Therapeutic Applications	Ref.
HA/HACC@DXMS&bFGF	Multifunctional dissolvable polysaccharide composite based on hyaluronic acid (HA) and hydroxypropyltrimethylammonium chitosan chloride (HACC) with dexamethasone (DXMS) and basic fibroblast growth factor (bFGF)	DXMS, bFGF	PDMS	Centrifuged at 12,000 rpm for 30 min	Oral ulcers healing	[59]
HA MNs containing CS-MoS_2_	Soluble microneedles based on hyaluronic acid (HA) with a nanocomposite of chitosan and molybdenum sulfide (CS-MoS_2_)	Chitosan (CS)/molybdenum sulfide (MoS_2_)	Silicone/PDMS	5 min under vacuum and centrifuged at 3000 rpm for 10 min, dried at room temperature for 24 h	Antibacterial	[60]
HA–Si MN	Fast-dissolving microneedles based on hyaluronic acid (HA) and in situ precipitated silica nanoparticles (Si) with insulin (pretest)	Insulin (pretest)	PDMS	Centrifuged (4000 rpm at 25 °C) for 10 min, dried at room temperature for 24 h	Diabetes	[61]
MTX-loaded HA-MN	Soluble microneedles based on hyaluronic acid (HA) loaded with methotrexate (MTX)	Methotrexate (MTX)	PDMS	Vacuum (∼0.08 MPa), dried at room temperature	Psoriasis	[62]
Rg3-MN	Soluble microneedles based on hyaluronic acid loaded with liposomes containing ginsenoside Rg3	Ginsenoside Rg3 liposome	PDMS	Centrifuged in a swinging bucket rotor at 3500 g for 15 min at 25 °C	Psoriasis	[63]
Cur-loaded Que-DA-oHA MN	An amphiphilic polymer-based material composed of quercetin (Que), dithiodipropionic acid (DA), and oligomeric hyaluronic acid (oHA), containing curcumin-loaded micelles	Curcumin (Cur)	-	Frozen at −20 °C for 6 h and melted. The freezing-thawing process repeated three times	Skin diseases	[64]
TA-HA/HP-β-CD	Soluble microneedles based on hyaluronic acid (HA) with hydroxypropyl-β- cyclodextrin (HP-β CD) containing triamcinolone acetonide (TA)	Triamcinolone acetonide (TA)	PDMS	Centrifuged at 4000 rpm for 5 min at 2 °C, centrifuged at 4000 rpm for 60 min at 25 °C, centrifuged at 2000 rpm for another 3 min, dried in a desiccator at room temperature for 72 h	Hypertrophic scar therapy	[65]
Shikonin HA MN	Soluble microneedles based on hyaluronic acid loaded with shikonin	Shikonin (an active component extracted from the root of *Lithospermum erythrorhizon*)	PDMS	Centrifuged (3200× *g*, 5 min), dried in fume hood at room temperature	Hypertrophic scar therapy	[66]
5-ALA-MN	Soluble microneedles based on hyaluronic acid with 5-aminolevulinic acid (5-ALA)	5-aminolevulinic acid (5-ALA)	PDMS	Centrifuged at 4000 rpm for 35 min (21 °C), dried in an oven at 40 ± 1 °C for 24 h	Precancerous skin lesions	[67]
5-Fu-ICGMPEG-PCL@HA MN	NIR-responsive hyaluronic acid-based microneedles integrated with monomethoxy-poly(ethylene glycol)-polycaprolactone (MPEG-PCL) nanoparticles loaded with 5-fluorouracil (5-Fu) and indocyanine green (ICG)	5-fluorouracil (5-Fu), indocyanine green (ICG)	PDMS	Centrifuged, dried overnight in an oven at 45 °C	Skin cancer therapy	[68]
DOX-T/MNs	Soluble hyaluronic acid-based microneedles integrated with transferomes (T) loaded with doxorubicin hydrochloride (DOX)	Doxorubicin hydrochloride (DOX)	PDMS	Centrifuged, dried at room temperature	Tumor metastasis therapy	[69]
DOX/DMNs	Soluble hyaluronic acid-based microneedles loaded with doxorubicin (DOX)	Doxorubicin (DOX)	PDMS	Vacuum for 30 min, dried in a ventilated place for 12 h	Cancer treatment	[70]
DOX/SMNs	Swellable microneedles based on methacrylated hyaluronic acid loaded with doxorubicin (DOX)	Doxorubicin (DOX)	PDMS	Vacuum for 30 min, dried in a ventilated place for 12 h	Cancer treatment	[70]
p53 DNA/IR820 MN	Soluble hyaluronic acid-based microneedles loaded with p53 expression plasmid (p53 DNA) and indocyanine green derivative (IR820)	p53 DNA, IR820	PDMS	Centrifuged at 5000 rpm for 15 min, dried overnight at 41 °C under vacuum	Synergistic combination of gene therapy and photothermal therapy of subcutaneous tumor	[71]
HA MN	Soluble hyaluronic acid-based microneedles	Hyaluronic acid (HA)	PDMS	Vacuum (~0.08 MPa), dried overnight in a sealed desiccator	Improving the permeability of skin tissues	[72]
DMNP loaded Hup A	Soluble hyaluronic acid-based microneedles containing dry huperzine A (Hup A) powder between layers of HA	huperzine A (Hup A)	PDMS	Centrifuged, dried overnight at room temperature	Alzheimer’s disease	[73]
HA MN	Soluble hyaluronic acid-based microneedles	Hyaluronic acid (HA)	PDMS	Vacuum for 45 min, dried overnight at room temperature	Safety evaluation	[74]
X-linked HA-NPs	Soluble microneedles based on crosslinked hyaluronic acid nanoparticles	Crosslinked hyaluronic acid nanoparticles (X-linked HA NPs)	PDMS	Centrifuged, dried at room temperature for 24 h	Sustained release test	[75]
RhB-loaded HA-MN	Soluble microneedles based on hyaluronic acid loaded with rhodamine B (RhB)	Rhodamine B (RhB)	PDMS	Centrifuged at 4000 rpm for 5 min, fan-dried for 48 h	Mechanical strength tests: the influence of HA molecular weight on the mechanical properties of HA-MNs; delivery efficiencies tests: transdermal delivery of rhodamine B (in vitro and in vivo)	[76]

**Table 3:** Characteristics of 3D printing technology in the formation of microneedles.

Technology	Material	Microneedle Type	Power Source	Ref.
Stereolithography (SLA)	Liquid Polymers	Solid, Hollow, Coated	UV Light	[96]
Selective Laser Sintering (SLS)	Metals, Polymers, Ceramics, and Thermoplastics	Solid, Biocompatible, Hollow	Laser Beam	[97]
Digital Light Processing (DLP)	Epoxides, Acrylates	Solid, Hollow, Coated, Biodegradable, Hydrogel	UV Light	[98]
Fused Deposition Modeling (FDM)	Thermoplastic Polymers, Metal, Glass	Solid, Biodegradable	Heat	[95]
Two-Photon Polymerization (2PP)	UV-curable acrylates, ceramics, resins.	Solid, Hollow	UV Light	[99]
Continuous Liquid Interface Production (CLIP)	UV-curable resins, acrylates	Solid, Coated, Hydrogel, Biodegradable	UV Light	[100]
